# Power Spectral Density Analysis of Purkinje Cell Tonic and Burst Firing Patterns From a Rat Model of Ataxia and Riluzole Treated

**DOI:** 10.15412/J.BCN.03080108

**Published:** 2017-01

**Authors:** Samira Abbasi, Ataollah Abbasi, Yashar Sarbaz, Mahyar Janahmadi

**Affiliations:** 1. Computational Neuroscience Laboratory, Department of Biomedical Engineering, Faculty of Electrical Engineering, Sahand University of Technology, Tabriz, Iran.; 2. Department of Mechatronics, School of Engineering- Emerging Technologies, University of Tabriz, Tabriz, Iran.; 3. Neuroscience Research Center, Shahid Beheshti University of Medical Sciences, Tehran, Iran.

**Keywords:** Cerebellar ataxias, Purkinje cells, Calcium spike, Sodium spike, Signal processing

## Abstract

**Introduction:**

Purkinje Cell (PC) output displays a complex firing pattern consisting of high frequency sodium spikes and low frequency calcium spikes, and disruption in this firing behavior may contribute to cerebellar ataxia. Riluzole, neuroprotective agent, has been demonstrated to have neuroprotective effects in cerebellar ataxia. Here, the spectral analysis of PCs firing in control, 3-acetylpyridine (3-AP), neurotoxin agent, treated alone and riluzole plus 3-AP treated were investigated to determine changes in the firing properties. Difference in the power spectra of tonic and burst firing was assessed. Furthermore, the role of calcium-activated potassium channels in the power spectra was evaluated.

**Methods:**

Analysis was performed using Matlab. Power spectral density (PSD) of PCs output were obtained. Peak frequencies were extracted from the spectrum and statistical comparisons were done. In addition, a multi-compartment computational model of a Purkinje cell was used. This computational stimulation allowed us to study the changes in the power spectral density of the PC output as a result of alteration in ion channels.

**Results:**

Spectral analysis showed that in the spectrum of tonic and burst firing pattern only high sodium frequency and low calcium frequency was seen, respectively. In addition, there was a significant difference between the frequency components of PCs firing obtained from normal, ataxia and riluzole treated rats. Results indicated that sodium firing frequency of normal, ataxic and treated PCs occurred in approximate frequency of 22.53±5.49, 6.46±0.23, and 31.34±4.07 Hz, respectively; and calcium frequency occurred in frequency of 4.22±2.02, 1.52±1.19, and 3.88±1.37 Hz, respectively. The simulation results demonstrated that blockade of calcium-activated potassium channels in the PC model changed the PSD of the PC model firing activity. This change was similar to PSD changes in ataxia condition.

**Conclusion:**

These alterations in the spectrum of PC output may be a basis for developing possible new treatment strategies to improve cerebellar ataxia.

## 1. Introduction

The cerebellar Purkinje cell is one of the most important neurons in the mammalian nervous system. Normal PCs spontaneously fire action potentials, and provide the single output of the cerebellar cortex (De Schutter & Bower, 1994a; De Schutter & Bower 1994b; Miyasho et al., 2001). Four representative Purkinje cell firing patterns were reported in the experimental studies: Simple spikes (contain high frequency sodium spikes), Na^+^ spike bursts (are separated by near-random pauses), trimodal state (includes a tonic segment of simple spikes, followed by a period of Ca^2+^ spike bursts, and finished with a quiescent period, in a highly cyclic pattern.), and bursts of Na^+^/Ca^2+^ spike bursts (separated by pauses) (Ze’ev et al., 2010).

The electrical behavior of PCs is complex and its general features are difficult to be appraised quantitatively. Nowadays, signal processing methods are used to analyze different aspects of biological phenomena, including neuronal firing behavior.

Recently, a singnal processing study (Ze’ev et al., 2010) proposed a spectral analysis of the Purkinje neuron output, as a combination of three inherent frequencies observed in its spectrum. These frequencies were due to the calcium spikes (1–15 Hz), sodium spikes (30–300 Hz) and the switching behavior between silent and firing states (below 1 Hz). The complex behavior of Purkinje cells could be defined by amplitude and frequency modulations of the frequency bands related to the switching behavior, sodium and calcium spikes. The Switching frequency was demonstrated for the first time by Ze’ev et al. in vitro condition (Ze’ev et al., 2010), however in vivo experiments showed similar slow oscillations between 0.039–0.078 Hz (Chen et al., 2009). Slow oscillations of firing and quiescence could be described as an astable mode, versus the known bistable mode in the PCs (Yartsev, Givon-Mayo, Maller, & Donchin, 2009). A new theory of Purkinje cell function using the terminology of electronic oscillator systems has also been defined which represent astable, bistable and monostable modalities (Abrams & Zhang, 2011).

Dysfunction or degeneration of PCs causes cerebellar ataxia. Cerebellar ataxia is a disease described by incoordination, instability of posture, disturbed gait, and intention tremor (Bird, 2014; Wallesch & Bartels, 1996). Unfortunately, the confirmed cure for cerebellar ataxia is still lacking. Available treatment try to reduce the severity of ataxias, therefore, it is necessary to diagnose it as early as possible (Abbasi et al., 2014). Currently neuroprotective agents are promising therapies for treatment of neurodegenerative diseases, such as cerebellar ataxia (Alviña & Khodakhah, 2010). Several experimental studies on animal models of ataxia (Janahmadi et al., 2009; Strupp et al., 2004; Weisz, Raike, Soria-Jasso, & Hess, 2005) demonstrated a significant neuroprotective effect for riluzole in ataxia. The lack of cure for cerebellar ataxia motivated us to use signal processing methods in this area, and development new approaches. Exploring the changes in the spectrum of Purkinje neurons output may be a basis for new perspectives on the treatment of cerebellar ataxia.

Changes in the electrical behavior of the PCs in ataxic condition could be studied by evaluating possible changes in the power spectral density (PSD) of their output signal (Abbasi et al., 2014). Therefore, this study investigated the power spectral density of the firing activity (tonic and burst) of PCs to further analyze changes in the intrinsic properties of ataxic PCs. By describing the PC output in terms of its frequencies, frequency description of the output of PCs from ataxic rats was analyzed and the frequency differences with respect to the normal condition were determined. To further analyze the PSD alterations, the PSD of PCs from riluzole plus 3-AP treated group was investigated. Moreover, the PSD of tonic and burst firing pattern in each group was examined separately. This study also investigated the role of the calcium-activated potassium channels blockade in the PSD of a PC output using a multi-compartment computational model of the Purkinje cell.

## 2. Methods

Analysis was performed using MATLAB (R2010a) software. The proposed structure consisted of three steps. In the first step power spectral density of PCs output were obtained. Then, in the second step, peak frequencies were extracted from the obtained spectrum and statistical comparisons were done. In the third step, the frequency domain of power spectra (0 to 100 Hz) was divided into 10 equal parts and the surface area under each interval was calculated.

### 2.1. Experimental data

This database includes PCs output of normal, 3-AP treated alone, and riluzole^+^ 3-AP treated rats. Animal model of ataxia was created using 3-AP which is a neurotoxin agent (Janahmadi et al., 2009). Numerous studies by utilizing the selective properties of 3-AP have evaluated the climbing fiber inputs to the cerebellum and the consequence of ablation of the inferior olive on Purkinje neurons (Gasbarri, Pompili, Pacitti, & Cicirata, 2003). Treatment with 3-AP altered electrophysiological properties of cerebellar Purkinje neurons. Therefore, the animals showed ataxia and lost their balance (Janahmadi et al., 2009). Spontaneous activity of PCs was recorded from the cell body using whole cell patch clamp recording under current clamp condition. About 300s of activity of each cell was recorded. These data and details of the recording methods were presented before (Janahmadi et al., 2009). The firing activity of 20 neurons in control condition, 19 cells from 3-AP treated rats, and 4 PCs from riluzole^+^ 3-AP treated group were used in this study. Segments of 60s recording were taken for analysis, and this 60s duration was arbitrarily chosen.

### 2.2. Power spectra

Spectral analysis is a potent tool in signal processing tasks and it has been used for processing of different biological signals (Sarbaz et al., 2012). To identify the frequency components of the PCs output, the PSD of each 60s recorded signal was computed, after subtracting the mean of the signal, to omit zero frequency component. In computing the PSD, a window equal to one-third of the signal length was used, while the original size of the signal was maintained by zero-padding of twice the size of the window.

A rectangular moving average filter applied on the spectrum to smooth the PSD. Application of such a filter to the spectrum significantly improved the next peak detection in the spectrum. Peaks detection in the spectrum was done both by taking the position of the maximum and by exploiting the property of the Hilbert transform (HT). The HT of a real signal x(t) is defined as:

$$\overset{⌢}{x}(t)=H[x(t)]=\frac{1}{\pi t}\times x(t)=\frac{1}{\pi }\int_{-\infty }^{\infty }\frac{x(Ʈ)}{t-Ʈ}dT$$

**Figure 1:**
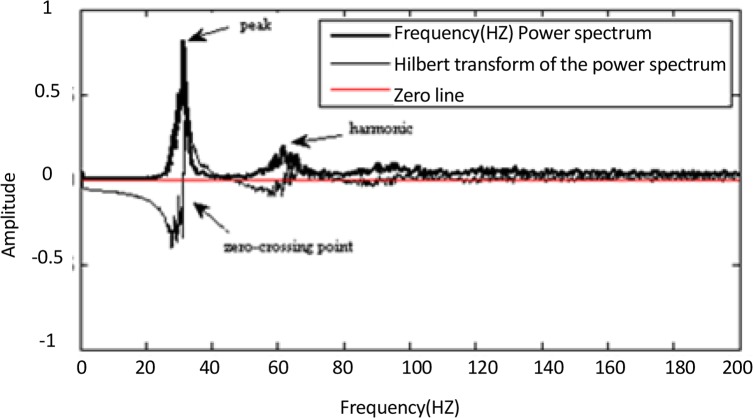
Illustration of peak detection method. The maximum value of power spectrum corresponds to the zero-crossing point (negative to positive transition) of the Hilbert transform.

It showed that positive zero-crossing (or negative-to-positive transition) points of the Hilbert transform of the x(t) correspond to locations of the peaks and the performance of the peak-finding was excellent (Manikandan & Soman, 2012). Therefore, the zero-crossings accompanied by negative to positive transition in the HT of the PSD were detected and used as guides to locate peaks in the spectrum (Figure 1). Harmonics in the PSD were seen, only the first (leftmost) peak in a series of equally-spaced peaks was considered. Data are presented as the Mean±SD.

Based on a spectral analysis of PCs behavior, frequencies of the peaks were collected for the spectrum of all cells and used to compare the cells’ electrophysiological behavior in order to demonstrate possible electrophysiological changes in ataxic condition and following riluzole treatment.

Frequencies of the peaks are appropriate features for differentiating normal cells from those that have been treated with 3-AP, and could be used in analysis of PCs output. For this purpose, we identified the sodium and calcium peak frequency of cells that may help determine the differences between ataxic and normal groups.

In addition to the peak detection, we analyzed also the surface area under the spectrum. Since the main frequency components were in the range of 0 to 100 Hz, We divided this range of the spectrum into smaller parts that may help for determining the differences between different patterns (Power spectra of tonic and burst firing were different) and different groups. Therefore, we divided the frequency spectrum into 10 equal parts and calculated the surface area under the spectrum curve in each part.

### 2.3. Statistical analysis

Lilliefors test (Lilliefors, 1967) was used to investigate whether peak frequencies had normal distribution. This test is suitable for small samples of data (Lilliefors, 1967). Then, statistical comparisons between groups were performed by Wilcoxon signed-rank test, and differences were considered significant if P<0.01.

### 2.4. Purkinje cell model

In this study, a PC model (De Schutter & Bower, 1994a; De Schutter & Bower 1994b) was used in order to explore the effect of ion channels blockade in the spectral analysis of the PC output. This model is a multi-compartmental Purkinje cell model with soma and active dendrites, and has been previously described in detail (De Schutter & Bower, 1994a; De Schutter & Bower 1994b). The model contains 10 ion channels consisting of fast sodium (NaF), persistent sodium (NaP), delayed rectifier potassium (Kdr), P-type calcium (CaP), T-type calcium (CaT), anomalous rectifier h-current (Kh), persistent potassium current (KM), A-type potassium (KA), BK calcium-activated potassium current (KC), K_2_ calcium-activated potassium current (K_2_). These ion channels were presented by using Hodgkin-Huxley model.

This model could fire spontaneously in the absence of synaptic inputs, also in the presence of synaptic inputs. This model contains 1474 spines, and each of these spines is inhibited and activated at the mean frequency of 1 and 28 Hz, respectively.

To investigate the firing activity of the neuron in the condition of blocking calcium-activated potassium channels, the maximum conductance of these ion channels was set to zero in the model, and its effect on firing activity of the PC was studied. The simulations were performed in the NEURON simulator (Version 7.1) (Hines & Carnevale, 1997).

## 3. Results

In our database, PCs displayed tonic and burst firing modes. Seven out of twenty normal cells had tonic firing while the rest thirteen had burst firing. In 3-AP treated group three out of nineteen cells showed tonic firing, while the rest sixteen neurons showed burst firing. Finally, two out of four cells in riluzole treated group had tonic and two showed burst firing.

By detecting peaks in the spectrum of the cells there were two frequency bands, one for calcium spiking and the other for sodium spiking. A tonic output displayed high frequency Na^+^ spikes and a burst output displayed low frequency Ca^2+^ spikes. Therefore, the sodium frequency band could be seen in PSD of PCs showing tonic firing and the calcium frequency was apparent in PSD of cells with burst activity.

**Figure 2:**
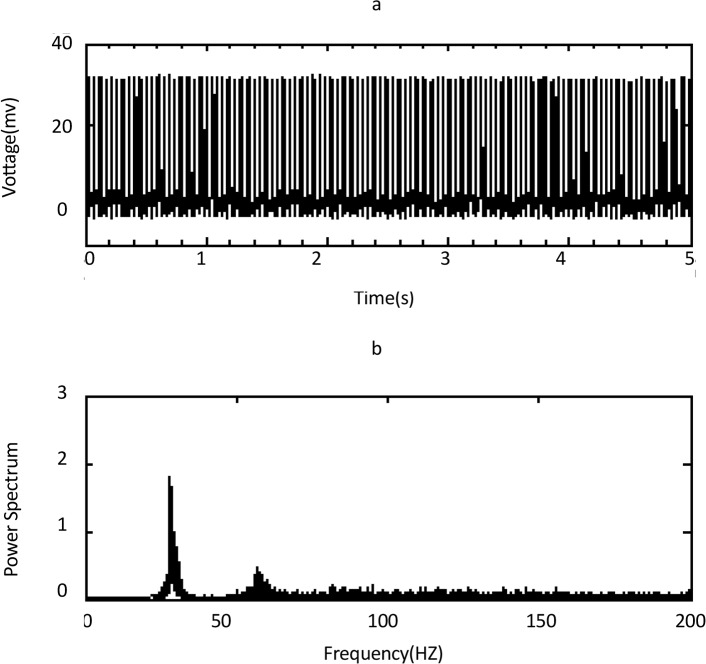
time and frequency domain of a normal Purkinje cell with tonic firing activity. a) Time domain, b) Frequency domain of panel a. The sodium peak occurs in frequency about 28.5 Hz. The second peak in about 57 Hz is harmonic of the first peak.

**Figure 3:**
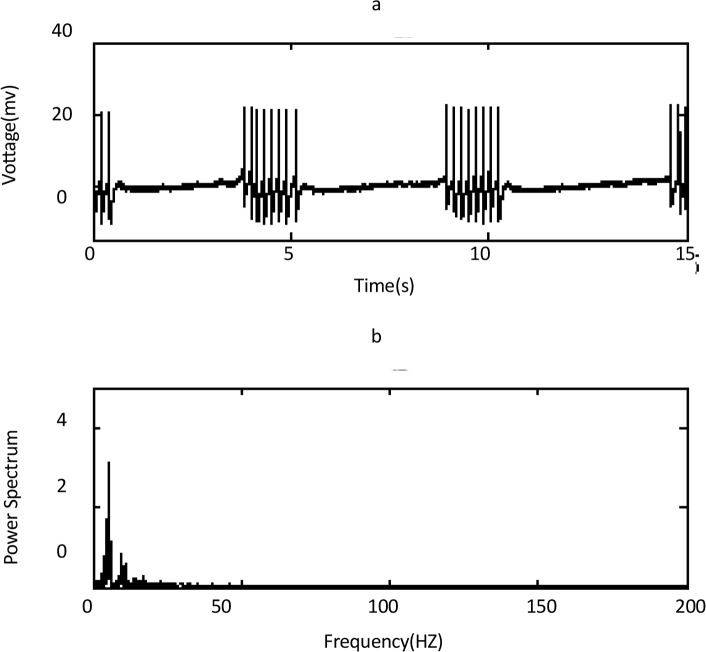
time and frequency domain of a normal Purkinje cell with burst firing activity A) time domain, B) frequency domain of panel a. The calcium peak occurs in frequency about 5.3 Hz. The second peak in frequency about 10.6 Hz is harmonic of the first peak.

The sodium frequency existed in the PSD of tonic firing in a range between 15.05–31.2 Hz (Figure 2), and calcium frequency existed in the PSD of burst firing in a range between 1.04–7.85 Hz for normal cells (Figure 3).

In normal cells (n=20) the frequency bands from the data were: Sodium: 22.53±5.49 Hz (n=7/20 with tonic behavior); Calcium: 4.22±2.02 Hz (n=13/20 with burst behavior) (Mean±SD).

The surface area under power spectra was measured in order to evaluate the differences between burst and tonic firings in each group, and differences between each group. In burst modes of activity, the main frequency components were in low frequency range as 70.24±15.32% of energy of signal was in the range of 0 to 10 Hz. The percentage of energy for tonic firing was 4.01±1.79.

Alterations in the firing activity of PCs from ataxic rats can be assessed by evaluating the possible changes in power spectral density of their output signals. Therefore, in this study, the power spectral densities of nineteen PCs of 3-AP treated group were evaluated. In this group of rats, the sodium frequency in the PSD of cells with tonic firing was in the range of 6.22 and 6.68 Hz (n=3/19). The calcium frequency existed in the PSD of burst firing in the range between 0.35 and 4.57 Hz (n=16/19). The mean frequencies of calcium and sodium spiking peaks were significantly (P<0.01) lower in comparison to those of obtained in normal cells (1.52±1.19 Hz in 3-AP-treated versus 4.22±2.02 Hz in normal group for calcium peaks and 6.46 ± 0.23 Hz in 3-AP treated versus 22.53±5.49 Hz in normal cells for sodium peaks).

In the output of Purkinje cells from ataxic group the main frequency components were in the low frequencies range. Energy of the signals from ataxic group with burst and tonic firing activity in the range of 0 to 10 Hz was 82.74±9.21, and 57.93±16.79 percent, respectively.

**Figure 4:**
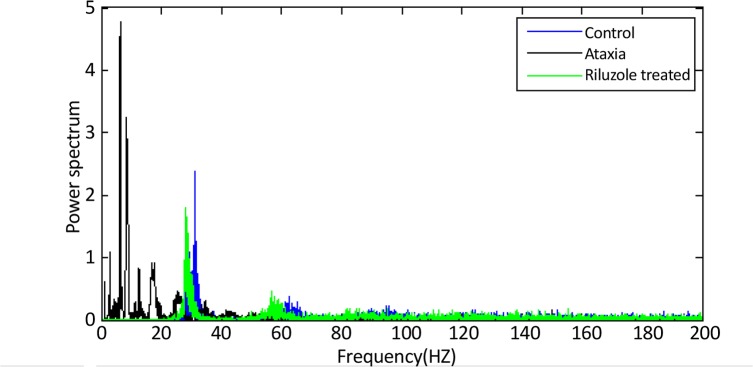
PSD of a tonic firing activity from control (blue), ataxia (black), and riluzole treated (green) PC. Frequency changed in ataxia condition, and treatment by riluzole could bring the frequency closer to the normal condition.

Our previous experimental studies (Janahmadi et al., 2009) showed that riluzole restored the normal firing activity in Purkinje cells from 3-AP plus riluzole treated rats. In the present study, Purkinje cells from riluzole treated along with 3-AP had sodium frequency of tonic firing about 31.34±4.07 Hz (n=2/4); the calcium frequency of burst was 3.88±1.37 Hz (n=2/4). These results are summarized in Table 1. In order to see differences in the spectrums, the PSD of a tonic firing activity from each group is shown in Figure 4.

**Table 1:** Sodium and calcium frequency of normal, ataxia and riluzole treated groups. Symbols ‘^*^’ and ‘^**^’ represent significant difference from normal group for sodium and calcium frequency, respectively (P<0.001). Symbol ‘^†^’ denotes significant difference between sodium and calcium frequency in the same group (P<0.001).

**Groups**	**Sodium Frequency in Tonic Behavior (Hz)**	**Calcium Frequency in Burst Behavior (Hz)**
Normal	22.53±5.49(n=7/20)^†^	4.22±2.02(n=13/20)^†^
3-AP treated	6.46±0.23(n=3/19)^*^	1.52±1.19(n=16/19)^**^
Riluzole plus 3-AP treated	31.34±4.07(n=2/4)^†^	3.88±1.37(n=2/4)^†^

**Figure 5:**
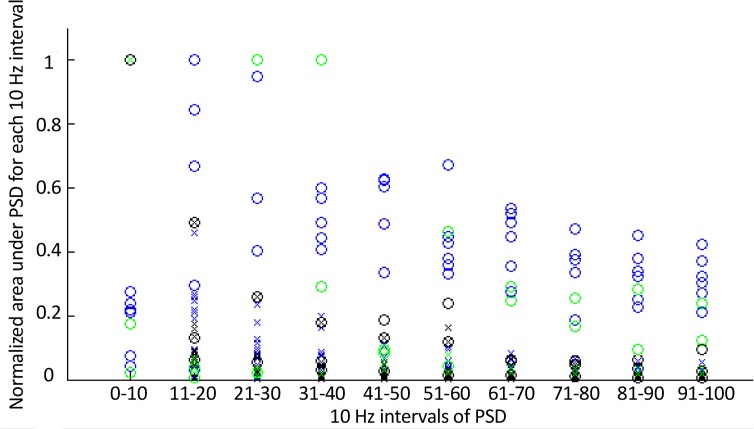
The normalized surface area under power spectra in 10 Hz intervals of normal (blue), ataxia (black), and riluzole treated (green) PCs. Symbols ‘o’ and ‘x’ represent tonic and burst firing activity of PCs, respectively.

The normalized surface area under power spectra for each part of spectrum is represented in Figure 5. Simulation of Purkinje neuron demonstrated firing at about 83±0.5 spike/s, while an initial membrane potential was −68 mV. These results were consistent with the measurements of the firing activity of the PCs in experimental studies. So, these results were validated. The spectral analysis of the PC model output showed that sodium peak occurred in frequency about 25.7 Hz. The second peak which was harmonic of the first peak was seen in the PSD. Simulations indicated that blockade of BK and K_2_ calcium-activated potassium currents changed the PSD of the PC model. In this condition the main frequency components were in the low frequencies range.

## 4. Discussion

Purkinje neurons play a significant role in maintaining balance and adjusting movement. They represent the only output of the cerebellar cortex, and modify the firing response of the deep cerebellar nuclei. In addition, their neuronal function impairment may cause ataxia. Since many neurological disorders have similar symptoms; sometimes, it is difficult to diagnose cerebellar ataxia. Some ataxias and related disorders may be treatable, if they are diagnosed early enough. Usually, the disease cannot be cured completely, but proper treatment can help the patient lead a normal life. There is growing interest in using computer-aided analysis to differentiate between normal and disease states (Abbasi et al., 2014). These complications encourage us to investigate the power spectral density of normal, ataxic and riluzole treated Purkinje cells and their differences.

We investigated also the difference between power spectra of tonic and burst firing activities. Power spectra analysis is a powerful tool in signal processing tasks. It has been useful in processing different biological signals, and also has proper applications in biological signal processing. Power spectra can be a proper tool for identifying the differences between normal and Purkinje cell output in ataxic condition and different patterns of activity. Alterations in the spectrum of Purkinje cell output obtained from ataxic group could possibly reflect the changes in ion channels activity. Ze’ev et al. have proposed a spectral analysis of the Purkinje neuron output, as a combination of three inherent frequencies observed in its spectrum that are due to the sodium spikes, calcium spikes and the switching behavior of the neuron between quiescence and firing modes (Ze’ev et al., 2010). They also described Purkinje neuron function as an oscillator (Abrams & Zhang, 2011; Ze’ev et al., 2012).

Our work here describes the normal, ataxic and riluzole treated Purkinje cells outputs in terms of its frequencies and tries to determine the differences of the normal, ataxia and treated group and tonic and burst firing activity by studying their power spectra. This approach was based on signal processing analysis; the power spectra of normal, ataxic and treated Purkinje cells output were computed. The results of ataxia group and its power spectra showed that there was a significant (P<0.01) difference between the frequency components among normal and ataxic groups. Neuroprotective effect of riluzole was able to almost restore the frequency components of Purkinje cells output in ataxic condition to the normal values.

Ze’ev et al. reported that in the spectrum of all Purkinje cells (n=33) outputs sodium frequency peak was observed and in some of them (n=16/33) calcium frequency peak was also seen. In addition, they did not separate tonic and burst patterns of PCs firing activity (Ze’ev et al., 2010). In this study, in the spectrum of the Purkinje cell output signals only the first harmonic (leftmost) in a series of equally-spaced peaks was chosen which showed PSD had one general peak. In the PSD of tonic Purkinje cell output, sodium frequency peak was seen, and in the PSD of burst firing calcium frequency peak was observed.

For ataxic condition related study was not found. But, here in ataxic condition the main frequency components were in the low frequency range. This was investigated by the surface area under the PSD. The evaluation of power spectra in two groups showed significant differences between the main peak of the sodium and calcium bands. Each of these features had shown to be statistically different for the normal and ataxia group. The sodium and calcium frequencies were significantly higher in normal group with respect to ataxia group. Riluzole treatment could restore the frequency component of Purkinje cells output in ataxic condition to the normal conditions (Figure 4).

The spectral changes in ataxia condition may reflect the changes in ionic currents. Thus, analysis of PSD of PC output may represent changes in the ionic currents, and channels might be involved in cerebellar ataxia. Previous experimental studies (Goudarzi et al., 2010; Walter, et al., 2006; Weisz et al., 2005) reported that in the animal models of ataxia, the main functional alteration was the impairment of Ca^2+^ spike bursts in Purkinje cells, so the enhancement of the burst firing could be an effective treatment for ataxia (Otis & Jen, 2006). It was suggested that calcium-activated potassium channels contribute to the Ca^2+^ generation (Yazdi et al., 2007). In addition, it was reported that riluzole could activate these channels (Alviña & Khodakhah, 2010; Conte Camerino at al., 2007). Therefore, alterations in the frequency components may be caused by change in calcium-activated potassium channels activity. Here, the power spectrum of the PC output in the condition of calcium-activated potassium channels blockade was investigated using a conductance based model of the PC. Results showed alterations similar to ataxia condition in the power spectrum. However, a comprehensive research is needed to evaluate the effects of different ion channels.

Proposed analysis can be used as a tool for helping the clinicians to better understanding the mechanism of ataxia and this difference may be a good feature for discrimination signals of these two groups which may be similar in the onset of disease; therefore, cerebellar ataxia will be known better.

In summary, the results of this study suggest that PSD features can effectively differentiate between Purkinje cell output in normal and ataxic condition. Proposed approach could result in a promising technique for computer-aided diagnosis of cerebellar ataxia. However, more detailed experimental and modeling data are needed to reveal underlying mechanisms exactly.
